# A Population-Based Cohort Study on the Ability of Acupuncture to Reduce Post-Stroke Depression

**DOI:** 10.3390/medicines4010016

**Published:** 2017-03-15

**Authors:** Shuo-Ping Tseng, Yu-Ching Hsu, Ching-Ju Chiu, Shang-Te Wu

**Affiliations:** 1 Department of Chinese Medicine, Tainan Municipal Hospital, Tainan 700, Taiwan; cfc54321@gmail.com; 2 Department of Chinese Medicine, Tainan Hospital, Ministry of Health and Welfare, Tainan 701, Taiwan; 3 Institute of Gerontology, College of Medicine, National Cheng Kung University, Tainan 701, Taiwan; 4 Department of Internal Medicine and Neurology, Kuo General Hospital, Tainan 700, Taiwan; wst31853@yahoo.com.tw

**Keywords:** post-stroke depression, acupuncture

## Abstract

**Objective:** Post-stroke depression (PSD) is common and has a negative impact on recovery. Although many stroke patients in Taiwan have used acupuncture as a supplementary treatment for reducing stroke comorbidities, little research has been done on the use of acupuncture to prevent PSD. Accordingly, our goal is to investigate whether using acupuncture after a stroke can reduce the risk of PSD. **Method:** This population-based cohort study examined medical claims data from a random sample of 1 million insured people registered in Taiwan. Newly diagnosed stroke patients in the period 2000–2005 were recruited in our study. All patients were followed through to the end of 2007 to determine whether they had developed symptoms of depression. A Cox proportional hazard model was used to estimate the relative risk of depression in patients after being diagnosed as having had a stroke, with a focus on the differences in those with and without acupuncture treatment. **Results:** A total of 8487 newly-diagnosed stroke patients were included in our study; of these, 1036 patients received acupuncture more than five times following their stroke, 1053 patients received acupuncture 1–5 times following their stroke and 6398 did not receive acupuncture. After we controlled for potential confounders (e.g., age, sex, insurance premium, residential area, type of stroke, length of hospital stay, stroke severity index, rehabilitation and major illness–related depression), we found that acupuncture after stroke significantly reduced the risk of depression, with a hazard ratio (HR) of 0.475 (95% CI, 0.389–0.580) in frequent acupuncture users and 0.718 (95% CI, 0.612–0.842) in infrequent acupuncture users, indicating that acupuncture may lower the risk of PSD by an estimated 52.5% in frequent users and 28.2% in infrequent users. **Conclusions:** After we controlled for potential confounders, it appears that using acupuncture after a stroke lowers the risk of depression. Additional strictly-designed randomized controlled trials are needed to better understand the specific mechanisms relating acupuncture to health outcomes.

## 1. Introduction

Strokes are the third leading cause of death and the most common cause of complex disability in Taiwan [1]. Many medical complications of stroke are common and often lead to poor clinical outcomes, such as depression, known as post-stroke depression (PSD). PSD has a high prevalence and a negative impact on stroke patients’ long-term survival and well-being [2]. Thus, it is important to reduce or prevent PSD in stroke survivors.

Many studies have evaluated various approaches to prevent PSD, including pharmacological therapy and psychotherapy. In some clinical trials, medicines such as Escitalopram and Duloxetine have shown some effectiveness in preventing PSD; however, most remedies had small treatment effect [3]. Other studies have also revealed that rehabilitation after a stroke may have positive effects in preventing PSD [4].

Traditional Chinese medicine (TCM) is classified as one form of complementary and alternative medicine (CAM) and is popular in Asian countries; for example, 52.7% of stroke patients receive both Chinese herbal remedies and acupuncture/traumatology treatment in Taiwan [5]. Some studies have reported that acupuncture may be effective in preventing PSD; however, such studies have so far been either too small or lacking big data analysis. If a case is to be made for the usefulness of acupuncture in relation to PSD, it is imperative to provide empirical evidence showing its positive effects on stroke patients’ psychological well-being.

Potential confounders related to PSD have been explored, including gender (female) [6], disabilities, comorbidities [7], stroke severity [8], pre-stroke depression [9], cognitive impairment after stroke, dysphagia [10], incontinence [10], anxiety, and social isolation at follow-up [10]. In order to evaluate the preventive effect of acupuncture after subjects have been diagnosed as having had a stroke, a more rigorous study must be performed. In response, the main purpose of this study was to investigate whether acupuncture treatment after stroke attack reduces the risk of PSD after empirically controlling for covariates during the observation period.

## 2. Materials and Method 

### 2.1. Source of Data

Our study used reimbursement claims data obtained from the National Health Insurance Research Dataset (NHIRD) in Taiwan. The NHIRD covers more than 99% of the population and has contracts with 97% of the hospitals and clinics in Taiwan [11]. The National Health Research Institute maintains and updates the NHIRD. The institute has publicly released a sub-dataset composed of claims data for 1,000,000 randomly selected insurance enrollees for research and administrative purposes. This random subgroup represents approximately 5% of the entire insured population in Taiwan. This sub-dataset, consisting of a longitudinal health insurance database for 2005 (LHID2005), was employed for this study after obtaining approval from the National Health Research Institute review committee. For data analysis, we retrieved information about patients’ characteristics and medical care records by linking ambulatory care visit claims, in-patient expenditures by admissions, and the registry for beneficiaries. Secondary data were collected and administered by the Taiwan National Institute of Family Planning (now the Bureau of Health Promotion, BHP), and approved by their IRB. All data analyzed in this study were anonymized.

### 2.2. Participants and End-Point

**Figure 1:**
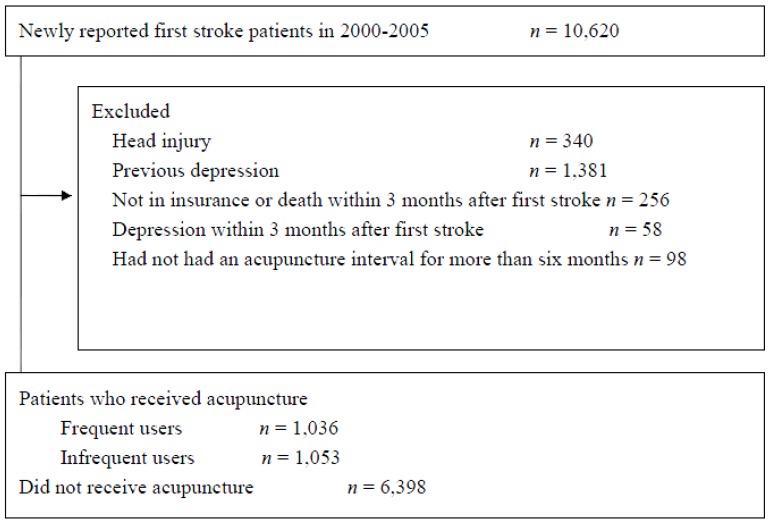
Flow chart showing details of subject recruitment from the National Health Insurance Research Dataset (NHIRD) of Taiwan for the years 2000 to 2005.

Patients who were newly diagnosed with a stroke (ICD-9-CM codes: 430–434, 436–437) between 2000 and 2005 were included in this study. We excluded patients who had had a head injury before 2000 (*N* = 340); who had suffered from depression before their stroke (*N* = 1381); who were not insured or had died within 3 months of suffering the stroke (*N* = 256); had sought ambulatory care for depression within the first 3 months following their stroke (*N* = 58); or had not had an acupuncture interval for more than six months (*N* = 98). The study criteria, the exclusion criteria, and the follow-up procedure are presented in Figure 1.

In Taiwan, TCM doctors must finish a training course in Chinese medicine and acupuncture and pass the national examination before they are certified to practice and, thereafter, qualified for filing NHI claims for acupuncture reimbursement. We classified the stroke patients into two groups based upon their acupuncture use or non-use after their stroke: (1) acupuncture users: those who received six or more or 1–5 acupuncture treatments after being diagnosed with a stroke from 2000 to 2007 (respectively *N* = 1036, 12.21%; and, *N* = 1053, 12.41%); these were respectively called frequent and infrequent users; and (2) acupuncture non-users: those who were defined as reporting no acupuncture received after their stroke from 2000 to 2007 (*N* = 6398, 75.39%).

The Diagnostic and Statistical Manual (DSM) IV categorizes PSD as a “mood disorder due to a general medical condition (i.e., stroke)” with certain depressive features serving as specifiers, for example, major depressive-like episodes, manic features, or mixed features [12]. The patients were linked to the ambulatory care visit claims and inpatient expenditures by admissions claims during the years 2000–2007 to identify possible treatment for depression. The follow-up ended on the date of depression diagnosis (diagnosed according to ICD-9-CM code 296, 309, or 311, or A-code A212 or A219) in outpatient care or on the date of censoring, which was either the date of withdrawal (including death) from the NHI program or the date of the follow-up end (i.e., 31 December 2007).

### 2.3. Covariates

The covariates considered in our analysis include socio-economic factors and covariates related to stroke severity or progress. The socio-economic factors include gender, age, living area (categorized as “urban area”, “satellite city”, and “rural area”) [13], and insurance premium (categorized as “<15,000 New Taiwan Dollars (NTD)”, and “≥15,000 NTD”).

The covariates related to stroke severity or progress include type of stroke (categorized as hemorrhagic, occlusion or others (e.g., transient ischemic attack)), length of hospital stay, rehabilitation after stroke in 3 months, comorbidities that are correlated to depression or disability, such as cancer (ICD-9-CM code: 140–208), arthritis or rheumatism (ICD-9-CM code: 714.0, 729.0), chronic obstructive pulmonary disease (ICD-9-CM code: 490–496), peripheral arterial disease (ICD-9-CM code: 440–449), diabetes (ICD-9-CM code: 250) chronic kidney disease (ICD-9-CM: 585) and ranking according to the stroke severity index (SSI). The SSI is an index that estimates a stroke’s severity by using six items listed in the hospitalization data in the NHI database [14], namely airway suction, bacterial sensitivity test, general ward stay, intensive care unit stay, nasogastric intubation, osmotherapy, and urinary catheterization.

### 2.4. Statistical Analysis

A chi-square test was used to compare differences in age, sex, residential area, insurance premium, type of stroke, length of hospital stay, and the aforementioned comorbidities between groups. A Kaplan–Meier analysis was performed for censored graft survival. To assess the independent effects of acupuncture on the risk of depression, we conducted a Cox proportional hazard regression analysis with age, sex, insurance premium, urbanization level, SSI, rehabilitation after stroke in three months, and selected comorbidities adjusted simultaneously in the model. We also adjusted the urbanization level to account for the urban rural difference in accessibility to medical care in Taiwan. All statistical analyses were performed using SAS (version 9.4, SAS Institute, Inc., Cary, NC, USA), in which a *p*-value < 0.05 was considered statistically significant.

## 3. Results 

### 3.1. Sample Characteristics

Characteristics of the newly-diagnosed stroke patients based on whether they are acupuncture users or non-users are presented in Table 1. A total of 8487 newly-diagnosed stroke patients were included in our study, 1036 patients defined as higher acupuncture users, 1053 patients defined as lower acupuncture users and 6735 patients defined as acupuncture non-users after stroke diagnosis. According to Table 1, stroke patients who received frequent acupuncture treatment were, on average, younger, more likely to live in an urban area, of higher economic status, more likely to have had a hemorrhagic stroke, and had more hospitalization days compared to nonusers. Both frequent or infrequent acupuncture users also received more rehabilitation treatment than non-users. The three participating groups also had different comorbid diseases, including rheumatoid arthritis, peripheral arterial disease, diabetes or hypertension. However, other factors such as gender, comorbidities with myocardial infarction, cancer, chronic kidney disease, chronic obstructive pulmonary disease or head traumatic injury were not statistically different between frequent and infrequent users and non-users. It was found that patients not using acupuncture treatment had a higher chance of being diagnosed with PSD or withdrawal (including death) from the NHI program than those in both the frequent and infrequent acupuncture groups. Over a 7-year follow-up, 110 patients (10.62%) from the frequent acupuncture group, 177 patients (16.81%) from the infrequent acupuncture group and 1551 non-users (24.24%) developed PSD.

### 3.2. Cox Proportional Hazard Regression Analysis

**Figure 2:**
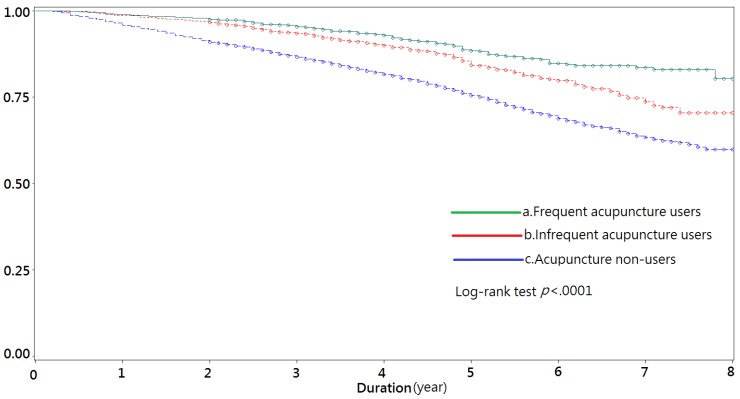
Kaplan–Meier Survival Curves of depression for comparing frequent acupuncture users, infrequent acupuncture users and non-users. a. Frequent acupuncture users: Subjects received six or more acupuncture treatments between 2000 and 2007 after stroke diagnosis; b. Infrequent acupuncture users: Subjects received 1–5 acupuncture treatments between 2000 and 2007 after stroke diagnosis; c. Acupuncture non-users: Subjects did not receive any acupuncture treatment between 2000 and 2007 after stroke diagnosis.

Figure 2 compares the Kaplan–Meier Survival Curves of depression between patients in the frequent and infrequent acupuncture treatment groups and the non-user group. As can be seen, patients with acupuncture treatment after stroke had a significantly lower risk of depression over the study period (*p* value for log-rank test ≤0.0001).

In Table 2, after controlling for potential confounders (e.g., age at diagnosis, gender, insurance premium level, living area, type of stroke, length of hospital stay, SSI, rehabilitation and major illness–related depression), acupuncture treatment after stroke appeared to significantly reduce the risk of PSD, with a hazard ratio (HR) of 0.475 (95% CI, 0.389–0.580) and 0.718 (95% CI, 0.612–0.842) for the frequent and infrequent acupuncture groups, respectively. In the multivariate analysis, females had a lower risk of depression than males (HR = 0.777; 95% CI, 0.705–0.856). Stroke patients with longer hospital stays, who are older, or have higher SSI tended to be at significantly greater risk of PSD after their stroke than the other patients in this study (HR = 1.098; 95% CI, 1.053–1.145; HR = 1.045; 95% CI, 1.040–1.050; HR = 1.210; 95% CI, 1.166–1.256, respectively). Comorbidity with myocardial infarction (HR =1.298; 95% CI, 1.080–1.559), cancer (HR = 1.423; 95% CI, 1.268–1.597), diabetes (HR = 1.136; 95% CI, 1.032–1.251), chronic kidney diseases (HR = 1.519; 95% CI, 1.345–1.715), chronic obstructive pulmonary disease (HR = 1.219; 95% CI, 1.097–1.355) or traumatic head injury (HR = 1.629; 95% CI, 1.335–1.988) also showed higher risk than those without these comorbidities. However, stroke patients living in urban areas, with peripheral arterial disease or hypertension had a lower risk of PSD after stroke diagnosis (HR = 0.836; 95% CI, 0.738–0.947; HR = 0.878; 95% CI, 0.781–0.986; HR = 0.844; 95% CI, 0.744–0.956, respectively). Meanwhile, economic status, rehabilitation, and presence of comorbid diseases with rheumatoid arthritis were not significantly more likely to be correlated with PSD risk. However, different risk of PSD development was found between hemorrhagic stroke and other types of stroke (HR = 0.796; 95% CI, 0.676–0.936). We further analyzed the effects of acupuncture treatment in developing PSD in hemorrhagic stroke and occlusion stroke (Table 3). The results suggest that occlusion-stroke patients receiving acupuncture treatment (either frequent or infrequent) would decrease the risk of developing PSD (HR = 0.499; 95% CI, 0.391–0.638; HR = 0.707; 95% CI, 0.578–0.865, respectively). In addition, patients with hemorrhagic stroke that received frequent acupuncture treatments appeared to minimize the risk of PSD (HR = 0.446; 95% CI, 0.283–0.702); however, those in the infrequent acupuncture group had no significant benefit over the non-users group (HR = 0.831; 95% CI, 0.577–1.196).

## 4. Discussion

Our results indicate that using acupuncture after stroke diagnosis lowers the risk of PSD by an estimated 52.5% (1–0.475) in frequent acupuncture users and 28.2% (1–0.718) in infrequent acupuncture users. Applying acupuncture to treat stroke complications is common in Taiwan, and to our knowledge, our study is the first to investigate the effects of acupuncture in preventing PSD in a large population-based cohort. Within a 7-year observation period, the results indicate that recently-diagnosed stroke patients using acupuncture after being diagnosed were likely to have a lower risk of PSD. The overall risk reduction is estimated to be about 30.6%.

Strokes may increase vulnerability to the development of depression through a variety of neurobiological mechanisms. Three possible explanations for the association between physical illness and depression include a coincidental relationship; a negative mood reaction to the physical consequences of the stroke; and a neurotransmitter imbalance as a result of cerebral damage caused by the stroke [15]. Based on these PSD mechanisms, our hypothesis is that using acupuncture after a stroke might improve stroke complications and result in the prevention of PSD. Studies suggest that acupuncture after a stroke may improve problems with pain [16], spasticity [16], physical functions [17], quality of life [18] and cognitive functions [19]. Our findings seem to support the hypothesis that acupuncture reduces complications caused by a stroke and thus lowers the risk of PSD.

However, the short-term or long-term effects of using acupuncture to prevent PSD are unknown. To address this issue, a sensitivity analysis controlling for acupuncture over a three-month or six-month period after a stroke diagnosis was performed (table not shown). The results did not show that receiving acupuncture offered benefits in terms of depression symptoms. Nevertheless, the study did reveal that TCM-use benefited patients with higher depressive symptoms by attenuating their worsening [20]. These results suggest that the preventive effect from a continuous acupuncture treatment period may vary widely after a stroke. Further randomized control trials between short-term or long-term follow-up times are needed for verification.

In addition, our study also explored potential confounders related to PSD. Females have a lower risk of depression than males, and the overall risk reduction is estimated to be about 22.1% in our study. Although these findings are similar to those found in [4], other research has reported that female stroke patients have a higher risk of PSD. However, our findings that stroke patients with advanced age [21], greater stroke severity [8] and compounded comorbidities [7] have a higher risk of PSD are consistent with much of the related prior literature. Be that as it may, a summed measure of comorbidities may ignore potentially important relationships between diseases and PSD. For example, our study revealed that stroke patients comorbid with myocardial infarction (HR = 1.301), cancer (HR = 1.414), diabetes (HR = 1.139), chronic kidney diseases (HR = 1.518), chronic obstructive pulmonary disease (HR = 1.218) or head traumatic injury (HR = 1.658) are at higher risk than their counterparts without these comorbidities. Furthermore, stroke patients with peripheral arterial disease or hypertension have a lower risk of depression after stroke diagnosis, while comorbid rheumatoid arthritis is not significantly correlated with PSD. Meanwhile, the benefit of 3-months rehabilitation was evaluated after stroke diagnosis in a previous study [4], but those results do not correspond with ours. This might be due to the short-term and long-term ability of acupuncture or rehabilitation to prevent PSD being unknown. Accordingly, it is suggested that future research include these comorbid factors or the long-term effects of rehabilitation to further our understanding in this line of research. In addition, there was little evidence that compared the risk of PSD between occlusion-stroke and hemorrhagic stroke. Our study provided some information to fill this research gap. The results also indicated that patients, with occlusion-stroke or hemorrhagic stroke, who received frequent acupuncture treatments, may minimize the risk of PSD (HR = 0.446–0.499).

Our study has some strong points. One is that it is the first population-based study to investigate acupuncture’s potential in preventing PSD based upon the real clinical conditions of stroke patients. A second is that acupuncture is a relatively low-cost treatment and, when performed by a TCM doctor, has none of the potential side effects associated with taking medicines. A third is that our study is an observational study based on reimbursement claims data obtained from the National Health Insurance Research Dataset (NHIRD). Compared to studies using questionnaires to detect depressive symptoms, our study reduced the potential of being confounded by the Hawthorne Effect. A fourth strong point is that stroke severity was evaluated in our study [14] to a greater degree than in other studies [4].

Nevertheless, this study still has some limitations. First, acupoints are hard to define using the NHI database; moreover, different acupuncturists use different acupoints in different patients, depending on the symptoms and severity of stroke complications. However, some clinical studies have shown that even sham-acupuncture may have treatment effects in some diseases. Thus, there may be a positive effect even without considering the location of acupoints in different individuals in our study. Second, disability is an important predictor of PSD, but the severity of disabilities is hard to define using the NHI database. However, we controlled for stroke severity and comorbidities to minimize the effects of the severity of disability. Third, potential confounders, such as brain lesion of stroke [22], cognitive impairment after stroke, dysphagia [10], incontinence [10], anxiety, and social isolation [10], are not included in the NHI database, and so may confound our research results. Accordingly, future work is needed to further this understanding. Fourth, patients in the acupuncture group may have also received other complementary treatments such as massage, herbs, and aromatherapy, among others. Thus, we may overestimate the treatment effect of acupuncture. As such, the effects of the more specific acupuncture mechanisms or interactions deserves future investigation when data is available. Moreover, we conducted the exclusion criteria for depression within the first 3 months following their stroke for the purpose of minimizing survivorship bias. Further sensitivity analysis (table not shown) was conducted and was found to be similar to the present results, suggesting the possible role of acupuncture in preventing PSD development. Fifth, because the benefits of medicines and psychotherapy or pre-acupuncture depression screening in preventing PSD were not available in our dataset or relevant to acupuncture treatment, we did not control for these variables in our study. Sixth, we cannot know the total number of dropouts and the main reasons for dropping out because our study used NHIRD, and so the reasons that patients choose not to continue acupuncture were based on individual will. Accordingly, further research examining the choice to dropout or remain is therefore warranted.

## 5. Conclusions

In conclusion, controlling for potential confounders, stroke patients who receive acupuncture may have a lower risk of PSD than those who do not. Moreover, acupuncture after a stroke may have a protective effect on depression, thus stemming further deterioration. Further strictly designed randomized controlled trials are needed to better understand the specific mechanisms of acupuncture and its impact on psychological health outcomes.

## Figures and Tables

**Table 1:** Demographic characteristics between frequent, infrequent acupuncture users and non-users in patients using acupuncture with newly diagnosed strokes from the 1-million enrollee random sample of the National Health Insurance Research Database (NHIRD) from 2000 to 2007 in Taiwan.

Characteristic	Frequent Acupuncture Users ^a^ (*N* = 1036, 12.21%)	Infrequent Acupuncture Users ^b^ (*N* = 1053, 12.41%)	Acupuncture Non-Users ^c^ (*N* = 6398, 75.39%)	*p* Value
Female (%)	444 (42.86)	425 (40.36)	2622 (40.98)	0.378
Age of diagnosis (Mean ± SD)	61.28 ± 13.19	61.77 ± 13.59	66.21 ± 14.35	<0.001
Follow-up time (year) (Mean ± SD)	4.67 ± 1.78	4.82 ± 1.76	4.28 ± 1.87	<0.001
Living area (%)				<0.001
Urban area	307 (30.10)	275 (26.52)	1471 (23.45)	
Satellite city	303 (29.71)	305 (29.41)	1668 (26.59)	
Rural area	410 (40.20)	457 (44.07)	3133 (49.95)	
Insurance income ranks (%) ^d,e^				<0.001
<15,000 NTD	320 (30.89%)	314 (29.82)	2379 (37.18%)	
≥15,000 NTD	716 (69.11%)	739 (70.18)	4019 (62.82%)	
Type of stroke				<0.001
hemorrhagic stroke	261 (25.19)	217 (20.61)	1288 (20.13)	
occlusion stroke	617 (59.56)	647 (61.44)	3923 (61.32)	
unknown	158 (15.25)	189 (17.95)	1187 (18.55)	
Hospitalization days				<0.001
≤7 days	458 (44.21)	536 (50.90)	3540 (52.20)	
8–14 days	253 (24.42)	298 (28.30)	1626 (25.52)	
15–21 days	111 (10.71)	74 (7.03)	541 (8.46)	
22–28 days	63 (6.08)	52 (4.94)	316 (4.94)	
≥28 days	151 (14.58)	93 (8.83)	575 (8.99)	
Comorbidities ^f^				
Rheumatoid arthritis (%)	91 (8.78)	83 (7.88)	440 (6.88)	0.018
Peripheral arterial disease (%)	265 (25.58)	234 (22.22)	1216 (19.01)	<0.001
Myocardial infarction (%)	50 (4.83)	54 (5.13)	278 (4.35)	0.312
Cancer (%)	152 (14.67)	173 (16.43)	919 (14.36)	0.400
Diabetes (%)	531 (51.25)	520 (49.38)	2989 (46.72)	0.003
Hypertension (%)	873 (84.27)	887 (84.24)	5160 (80.65)	<0.001
Chronic kidney diseases (%)	112 (10.81)	110 (10.45)	767 (11.99)	0.140
Chronic obstructive pulmonary diseases (%)	554 (53.47)	572 (54.32)	3592 (56.14)	0.070
Head traumatic injury (%)	23 (2.22)	31 (2.94)	231 (3.33)	0.055
Rehabilitation ^g^	390 (37.64)	295 (28.02)	1126 (17.60)	<0.001
SSI (Mean ± SD) ^h^	−0.16 ± 1.39	−0.35 ± 1.22	−0.14 ± 1.40	<0.001
Censor after stroke ^i^				
Yes	110 (10.62)	177 (16.81)	1551 (24.24)	<0.001

Abbreviation: NHIRD = National Health Insurance Research Database; SD = standard deviation; NTD = New Taiwan Dollar. ^a^ Frequent acupuncture users: Subjects received six or more acupuncture treatments between 2000 and 2007 after stroke diagnosis; ^b^ Infrequent acupuncture users: Subjects received 1–5 acupuncture treatments between 2000 and 2007 after stroke diagnosis; ^c^ Acupuncture non-users: Subjects did not receive any acupuncture treatment between 2000 and 2007 after stroke diagnosis; ^d^ The income-related insurance payment category set by the Bureau of National Health Insurance in Taiwan; ^e^ 1 US $ = 30 NTD (New Taiwan Dollars); ^f^ Comorbidities = Medical illness, including rheumatoid arthritis, peripheral arterial disease, myocardial infarction, cancer, diabetes, hypertension, chronic kidney diseases, chronic obstructive pulmonary disease, and head traumatic injury, which are related to acupuncture use and depression; ^g^ Rehabilitation: had rehabilitation in the 3 months after stroke; ^h^ SSI = Stroke severity index; ^i^ Censor after stroke: Subjects sought ambulatory care for depression in outpatient care after stroke diagnosis between 2000 and 2005 or withdrew (including death) from the NHI program in 2000–2007.

**Table 2:** Multivariable adjusted hazard ratios of covariates for depression.

Characteristic	Hazard Ratio ^a^	*p* Value	95% CI	
Acupuncture users (ref: Acupuncture non-users ^d^)				
Frequent acupuncture users ^b^	0.475	<0.001	0.389	0.580
Infrequent acupuncture users ^c^	0.718	<0.001	0.612	0.842
Female	0.777	<0.001	0.705	0.856
Age of diagnosis	1.045	<0.001	1.040	1.050
Living area (ref: Rural area)				
Urban area	0.836	0.005	0.738	0.947
Satellite city	0.966	0.548	0.861	1.082
Insurance income (ref: <15,000 NTD) ^e,f^				
≥15,000 NTD	1.082	0.128	0.978	1.198
Type of stroke (ref: unknown)				
hemorrhagic stroke	0.796	0.006	0.676	0.936
occlusion stroke	0.911	0.131	0.808	1.028
Hospitalization days ^g^	1.098	<0.001	1.053	1.145
Comorbidities				
Rheumatoid arthritis	0.998	0.989	0.833	1.195
Peripheral arterial disease	0.878	0.029	0.781	0.986
Myocardial Infarction	1.298	0.005	1.080	1.559
Cancer	1.423	<0.001	1.268	1.597
Diabetes	1.136	0.009	1.032	1.251
Hypertension	0.844	0.008	0.744	0.956
Chronic kidney diseases	1.519	<0.001	1.345	1.715
Chronic obstructive pulmonary diseases	1.219	<0.001	1.097	1.355
Head traumatic injury	1.629	<0.001	1.335	1.988
Rehabilitation ^h^ (ref: no rehabilitation)	0.929	0.221	0.825	1.045
SSI i	1.210	<0.001	1.166	1.256

Abbreviation: SD = standard deviation; NTD = New Taiwan Dollar; CI = confidence interval; ref = reference. ^a^ Hazard ratio (95% confidence interval) was adjusted for all listed variables in the table; ^b^ Frequent acupuncture users: Subjects received six or more acupuncture treatments between 2000 and 2007 after stroke diagnosis; ^c^ Infrequent acupuncture users: Subjects received 1–5 acupuncture treatments between 2000 and 2007 after stroke diagnosis; ^d^ Acupuncture non-users: Subjects did not receive any acupuncture treatment between 2000 and 2007 after stroke diagnosis; ^e^ The income-related insurance payment category set by the Bureau of National Health Insurance in Taiwan; ^f^ 1 US $ = 30 NTD(New Taiwan Dollars); ^g^ Hospitalization days treated as continuous variable (≤7, 8–14, 15–21, 22–28 and ≥28 days); ^h^ Rehabilitation: had rehabilitation in the 3 months after stroke; ^i^ SSI = Stroke severity index.

**Table 3:** Multivariable adjusted hazard ratios of covariates for depression between hemorrhagic stroke and occlusion stroke.

Characteristic ^e^	Hemorrhagic Stroke				Occlusion Stroke			
	Hazard Ratio ^a^	*p* Value	95% CI		Hazard Ratio ^a^	*p* Value	95% CI	
Acupuncture users (ref: Acupuncture non-users ^d^)								
Frequent acupuncture users ^b^	0.446	<0.001	0.283	0.702	0.499	<0.001	0.391	0.638
Infrequent acupuncture users ^c^	0.831	0.318	0.577	1.196	0.707	<0.001	0.578	0.865

Abbreviation: SD = standard deviation; NTD = New Taiwan Dollar; CI = confidence interval; ref = reference. ^a^ Hazard ratio (95% confidence interval) was adjusted for all listed variables in the table; ^b^ Frequent acupuncture users: Subjects received six or more acupuncture treatments between 2000 and 2007 after stroke diagnosis; ^c^ Infrequent acupuncture users: Subjects received 1–5 acupuncture treatments between 2000 and 2007 after stroke diagnosis; ^d^ Acupuncture non-users: Subjects did not receive any acupuncture treatment between 2000 and 2007 after stroke diagnosis; ^e^ Covariates including gender, age of diagnosis, living area, insurance income, hospitalization days, comorbidities (such as cancer, arthritis or rheumatism, chronic obstructive pulmonary disease, peripheral arterial disease, diabetes, chronic kidney disease), rehabilitation and the stroke severity index (SSI) were controlled.
